# Epidemiology and Molecular Identification and Characterization of *Mycoplasma pneumoniae*, South Africa, 2012–2015

**DOI:** 10.3201/eid2403.162052

**Published:** 2018-03

**Authors:** Maimuna Carrim, Nicole Wolter, Alvaro J. Benitez, Stefano Tempia, Mignon du Plessis, Sibongile Walaza, Fahima Moosa, Maureen H. Diaz, Bernard J. Wolff, Florette K. Treurnicht, Orienka Hellferscee, Halima Dawood, Ebrahim Variava, Cheryl Cohen, Jonas M. Winchell, Anne von Gottberg

**Affiliations:** National Institute for Communicable Diseases of the National Health Laboratory Service, Johannesburg, South Africa (M. Carrim, N. Wolter, S. Tempia, M. du Plessis, S. Walaza, F. Moosa, F.K. Treurnicht, O. Hellferscee, C. Cohen, A. von Gottberg);; University of the Witwatersrand, Johannesburg (N. Wolter, M. du Plessis, S. Walaza, E. Variava, C. Cohen, A. von Gottberg);; Centers for Disease Control and Prevention, Atlanta, Georgia, USA (A.J. Benitez, S. Tempia, M.H. Diaz, B.J. Wolff, J.M. Winchell);; Pietermaritzburg Metropolitan Hospitals, Pietermaritzburg, South Africa (H. Dawood);; Centre for the AIDS Programme of Research in South Africa, Pietermaritzburg (H. Dawood);; University of KwaZulu-Natal, Pietermaritzburg (H. Dawood);; Klerksdorp-Tshepong Hospital Complex, Klerksdorp, South Africa (E. Variava);; Perinatal HIV Research Unit, Johannesburg (E. Variava)

**Keywords:** Mycoplasma pneumoniae, community-acquired pneumonia, South Africa, severe respiratory illness, influenza-like illness, real-time PCR, macrolide susceptibility, molecular characterization, bacterial infection, bacteria, respiratory infections, antimicrobial resistance

## Abstract

During 2012–2015, we tested respiratory specimens from patients with severe respiratory illness (SRI), patients with influenza-like illness (ILI), and controls in South Africa by real-time PCR for *Mycoplasma pneumoniae*, followed by culture and molecular characterization of positive samples. *M. pneumoniae* prevalence was 1.6% among SRI patients, 0.7% among ILI patients, and 0.2% among controls (p<0.001). Age <5 years (adjusted odd ratio 7.1; 95% CI 1.7–28.7) and HIV infection (adjusted odds ratio 23.8; 95% CI 4.1–138.2) among *M. pneumonia*–positive persons were associated with severe disease. The detection rate attributable to illness was 93.9% (95% CI 74.4%–98.5%) in SRI patients and 80.7% (95% CI 16.7%–95.6%) in ILI patients. The hospitalization rate was 28 cases/100,000 population. We observed the macrolide-susceptible *M. pneumoniae* genotype in all cases and found P1 types 1, 2, and a type 2 variant with multilocus variable number tandem repeat types 3/6/6/2, 3/5/6/2, and 4/5/7/2.

In 1986, ≈4 million deaths were attributed to pneumonia in children <5 years old globally (*1*). This number declined to 1.2 million by 2011, largely because of interventions such as antimicrobial drugs and vaccination against leading pneumonia-causing pathogens (*1*). Despite this decline, pneumonia remains a major cause of illness and death globally, especially in children <5 years old (*2*).

*Mycoplasma pneumoniae* causes respiratory illness and pneumonia with estimated prevalence ranging 2%–35%, depending on the identification method, the study period, and the population under investigation (*3*–*5*). Data from 21 countries showed *M. pneumoniae* to be the most common atypical pneumonia-causing bacterium, responsible for ≈12% of community-acquired pneumonia during 1996–2004 (*6*). *M. pneumoniae* epidemics have been reported to occur in cycles of 3–5 years (*5*,*7*). Persons infected with *M. pneumoniae* are treated with macrolides or azalide antibiotics (*8*); however, because of excessive and inappropriate use of antibiotics, macrolide resistance is increasing (*9*–*11*).

Characterization of strains is important for outbreak investigations to understand disease epidemiology and to identify type switching that might occur because of population immune pressure. *M. pneumoniae* is typically characterized by typing the P1 adhesion molecule gene sequence, which distinguishes the 2 P1 types (*12*), or by using multilocus variable-number tandem-repeat analysis (MLVA), which is more discriminatory than P1 typing (*13*).

The prevalence of *M. pneumoniae* in South Africa is unknown because of the low availability of reliable tests and because clinicians rarely request testing. Here we describe the prevalence, epidemiology, molecular characteristics, and antimicrobial resistance properties of *M. pneumoniae* among patients with mild and severe respiratory illness in South Africa.

## Materials and Methods

### Study Design

We enrolled patients and asymptomatic persons during June 2012–May 2015 as part of 2 surveillance programs (1 for severe respiratory illness [SRI] and 1 for influenza-like illness [ILI]). SRI surveillance was conducted at 2 sentinel sites, Edendale Hospital in KwaZulu Natal Province and Klerksdorp-Tshepong Hospital Complex in North West Province. Patients enrolled in SRI surveillance were those hospitalized with clinical signs and symptoms of lower respiratory tract infection (LRTI), regardless of symptom duration. We included children 2 days to <3 months old who had physician-diagnosed sepsis or acute LRTI, children 3 months to <5 years old with physician-diagnosed LRTI, and persons >5 years old who met the World Health Organization case definition for LRTI (sudden onset of fever [temperature >38°C] or reported fever, cough or sore throat, and shortness of breath or difficulty breathing [*14*]).

ILI patients were outpatients who were seen at 2 primary health care clinics serving the 2 SRI sentinel sites. Patients were considered to have ILI if they had an acute fever of >38°C or a self-reported fever within the last 7 days and either a cough or sore throat. Asymptomatic persons included those who were seen at the same primary health care clinics and had no history of respiratory illness, diarrheal illness, or fever in the preceding 14 days. For asymptomatic persons, we aimed to enroll 1 HIV-infected and 1 HIV-uninfected person weekly in each clinic within the following age categories: 0–1, 2–4, 5–14, 15–54, and >55 years.

We obtained demographic and clinical information from all enrollees by using a standardized questionnaire. We reviewed hospital records of SRI patients to assess disease progression and outcome.

### Specimen Collection

We collected combined nasopharyngeal and oropharyngeal swabs from >5 year-old persons and nasopharyngeal aspirates from <5 year-old persons (nasopharyngeal specimens) and placed the specimens in universal transport medium (Copan Italia, Brescia, Italy). We collected induced or expectorated sputum from SRI patients only. HIV status was determined as part of standard care or by using anonymized-linked dried blood spot testing for consenting enrollees (PCR for children <18 months old and ELISA for persons >18 months old [*15*]). We tested nasopharyngeal specimens for 10 respiratory viruses (influenza types A and B, adenovirus, enterovirus, rhinovirus, human metapneumovirus, respiratory syncytial virus, and parainfluenza virus types 1–3) by using an in-house multiplex real-time reverse transcription PCR (*16*).

### Detection of *M. pneumoniae*

We extracted DNA from 200 µL of nasopharyngeal specimen and digested sputum by using the MagNA Pure 96 instrument (Roche Diagnostics, Mannheim, Germany) with the DNA and Viral NA SV kit (Roche Diagnostics). We performed an in-house multiplex real-time PCR for the detection of *M. pneumoniae, Chlamydia* (*Chlamydophila*) *pneumoniae*, and *Legionella* spp., with human ribonuclease P gene serving as an internal control, as previously described (*17*). A positive *M. pneumoniae* patient was defined as a patient having a positive PCR result with a cycle threshold value <45 for *M. pneumoniae* on the nasopharyngeal specimen, sputum specimen, or both.

### Culture and Molecular Characterization

We detected 82 cases of PCR-positive *M. pneumoniae* during study periods 1 (June 2012–May 2013) and 2 (June 2013–May 2014) and performed culture and molecular characterization retrospectively on 77 (94%) samples. Culture and further characterization could not be performed for 5 cases because of insufficient specimens.

We inoculated *M. pneumoniae*–positive specimens in SP4 medium (Thermo Fisher Scientific, Waltham, Massachusetts, USA) and incubated them at 37°C in 5% CO_2_ for up to 10 days. Growth was indicated by a color change from red to orange, without turbidity. We performed macrolide susceptibility analysis by using real-time PCR followed by high-resolution melt-curve (HRM) analysis by means of the Rotor-Gene Q6000 system (QIAGEN, Hilden, Germany), according to previously described methods (*18*).

We performed P1 genotyping by using real-time PCR targeting the 1900-bp region of the P1 gene, followed by HRM analysis using the Rotor-Gene Q6000 system according to previously described methods (*12*). We also performed MLVA typing on the same specimens by using 5 variable-number tandem-repeat loci (Mpn1, Mpn13, Mpn14, Mpn15, and Mpn16), as described by Dégrange et al. (*13*). However, for analysis, we used the 4-loci nomenclature as described by Sun et al. (*19*) because of the instability of the Mpn1 locus (*20*).

### Statistical Analysis

We used the χ^2^ or Fisher exact test for comparison of categorical variables. We used unconditional logistic regression to estimate the attributable fraction (AF) of *M. pneumoniae*–associated hospitalization and outpatient consultation by comparing the *M. pneumoniae* detection rate among SRI or ILI patients to that of controls. The AF was estimated from the odds ratio (OR) obtained from the regression models.

Among SRI patients, we estimated the AF for patients positive on nasopharyngeal specimens only as well as for patients positive on both nasopharyngeal and sputum specimens. We adjusted all estimates for age, HIV status, underlying medical conditions other than HIV infection, and co-infections with the 10 respiratory viruses investigated in this study.

In addition, we used unconditional logistic regression to assess factors associated with *M. pneumoniae*–associated SRI hospitalization by comparing the characteristics of *M. pneumoniae*–positive SRI patients with those of *M. pneumoniae*–positive ILI patients. For the multivariable model, we assessed all variables that were significant at p<0.2 on univariate analysis and dropped nonsignificant factors (p>0.05) with manual backward elimination. We assessed pairwise interactions by inclusion of product terms for all variables remaining in the final multivariable additive model. We performed the analysis by using Stata 14 (StataCorp LLC, College Station, Texas, USA). Seasonality and periodicity were assessed over 3 periods (period 1, June 2012–May 2013; period 2, June 2013–May 2014; and period 3, June 2014–May 2015).

### Calculation of Rates of *M. pneumoniae*–Associated SRI Hospitalization

We estimated the overall and age-specific rates of *M. pneumoniae*–associated SRI hospitalizations (per 100,000 population) by using the number of SRI hospitalizations and adjusting for nonenrollment (e.g., refusals to participate and no enrollment on weekends [*21*]) and healthcare-seeking behavior during 2013–2014. For all calculations, we assumed that the *M. pneumoniae* detection rate among persons tested and not tested was the same within age groups. We obtained age- and year-specific population denominators from projections of the 2011 census data (*21*), and we obtained age- and year-specific HIV prevalence in the study population from the projections of the Thembisa model (*22*).

We calculated 95% CIs for all estimated rates by using bootstrap resampling of all parameters included in the estimation over 1,000 replications. The upper and lower limits of the 95% CI were the 2.5th and 97.5th percentile of the estimated values from the bootstrapped datasets, respectively.

## Results

### Study Population

During June 2012–May 2015, we enrolled 11,391 persons, of whom 10,194 (89.5%) had specimens collected that were tested for *M. pneumoniae*. Of these specimens, 4,703 (46.1%) were from SRI patients, 3,721 (36.5%) were from ILI patients, and 1,770 (17.4%) were from controls. Among the SRI patients, 2,390 (50.8%) had a nasopharyngeal specimen tested only, 207 (4.4%) had sputum tested only, and 2,106 (46.8%) had both specimen types tested.

Among persons for whom age was known, children <5 years old accounted for 35.8% (1,678/4,687) of SRI patients, 30.7% (1,142/3,716) of ILI patients, and 35.2% (662/2,767) of controls. HIV status was known for 86.5% (8,815/10,194) of enrollees. HIV prevalence was 54.2% (2,117/3,902) among SRI patients, 28.8% (940/3,261) among ILI patients, and 42.7% (705/1,652) among controls (owing to enrollment criteria of controls) (p<0.001). Among SRI and ILI patients, HIV prevalence was lowest among infants <1 year old (SRI patients, 12.3% [99/805]; ILI patients, 2.5% [9/360]) and highest among persons 25–44 years old (SRI patients, 90.6% [1,180/1,303]; ILI patients, 59.0% [588/997]).

### Detection Rate of *M. pneumoniae*

Overall, we detected *M. pneumoniae* in 1.0% (103/10,194) of persons tested; this rate was 1.6% (73/4,703) among SRI patients, 0.7% (27/3,721) among ILI patients, and 0.2% (3/1,770) among controls (p<0.001). We also compared detection rates by age and HIV status (Table 1). Among patients with SRI, the detection rate of *M. pneumoniae* differed by specimen type (1.1% [49/4,496] in nasopharyngeal specimens vs. 1.7% [39/2,313] in sputum; p = 0.04). Among the 2,106 SRI patients with *M. pneumoniae* results available for both specimen types, 15 (0.7%) patients tested positive on both specimens, 10 (0.5%) tested positive on nasopharyngeal specimen only, 20 (0.9%) tested positive on sputum only, and 2,061 (97.9%) tested negative on both specimen types. Of *M. pneumoniae*–positive patients with known outcome, 98% (97/99) survived and 2% (2/99) died. Both patients that died were adults admitted for SRI and were receiving treatment for tuberculosis with no other respiratory virus identified. One of these patients was HIV-positive, and HIV status for the other patient was unknown.

**Table 1:** *Mycoplasma pneumoniae* detection rate, by age and HIV status, among inpatients with SRI, outpatients with ILI, and controls, Klerksdorp and Pietermaritzburg, South Africa, June 2012–May 2015*

Characteristic	No. positive/no. tested (%)		
	SRI	ILI	Controls
Total	73/4,703 (1.6)	27/3,721 (0.7)	3/1,770 (0.2)
Age group, y			
<1	19/1,067 (1.8)	3/397 (0.8)	0/249 (0.0)
1–4	19/611 (3.1)	8/745 (1.1)	0/373 (0.0)
5–24	9/383 (2.3)	8/1,085 (0.7)	3/505 (0.6)
25–44	16/1,492 (1.1)	6/1,075 (0.6)	0/271 (0.0)
45–64	7/885 (0.8)	1/360 (0.3)	0/258 (0.0)
>65	3/249 (1.2)	1/54 (1.9)	0/111 (0.0)
HIV status			
Negative	30/1,785 (1.7)	21/2,231 (0.9)	2/947 (0.2)
Positive	32/2,117 (1.5)	2/940 (0.2)	0/705 (0.0)

*ILI, influenza-like illness; SRI, severe respiratory illness.

We observed no seasonality (Figure). However, we detected a significantly higher rate of *M. pneumoniae* in periods 1 and 2 compared with period 3 (period 1, 1.4% [50/3,651] vs. period 2, 1.2% [35/2,846] vs. period 3, 0.5% [18/3,594]; p<0.001).

**Figure:**
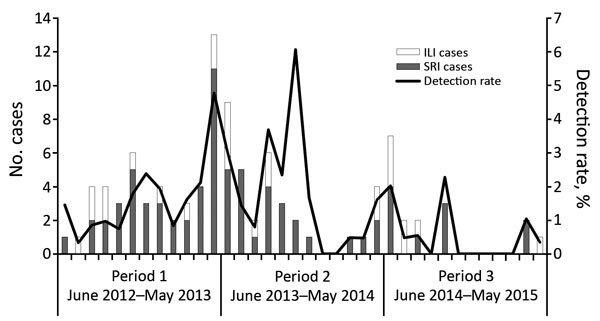
Number of *Mycoplasma pneumoniae*–positive cases and detection rate among inpatients with SRI and outpatients with ILI (N = 8,424), by month and period, Klerksdorp and Pietermaritzburg, South Africa, June 2012–May 2015. ILI, influenza-like illness; SRI, severe respiratory illness.

Overall, we detected *M. pneumoniae* along with another virus or other atypical pneumonia-causing bacteria in 65% (62/103) of patients. *M. pneumoniae* was co-detected with rhinovirus most frequently (58% [36/62]), followed by adenovirus (43% [27/62]), respiratory syncytial virus (18% [11/62]), influenza virus (13% [8/62]), human metapneumovirus (8% [5/62]), enterovirus (6% [4/62]), parainfluenza virus (5% [3/62]), and *C. pneumoniae* (3% [2/62]).

### AF of *M. pneumonia*e–Associated Hospitalization and Outpatient Consultation

The AF of *M. pneumoniae* detection to illness using nasopharyngeal specimens only for patients with ILI was 80.7% (95% CI 16.7%–95.6%) and for patients with SRI was 90.1% (95% CI 58.3%–97.7%). The AF of *M. pneumoniae* detection to illness for patients with SRI using both nasopharyngeal and sputum specimens was 93.9% (95% CI 74.4%–98.5%).

### Factors Associated with Hospitalization among *M. pneumoniae* PCR-Positive Patients

On multivariable analysis, we compared *M. pneumoniae*–positive SRI patients to *M. pneumoniae*–positive ILI patients. Factors associated with increased risk of *M. pneumoniae*–associated hospitalization were age <5 years compared with >5 years (adjusted odds ratio [aOR] 7.1; 95% CI 1.7–28.7); HIV infection (aOR 23.8; 95% CI 4.1–138.2); and duration of symptoms >4 days (aOR 3.8; 95% CI 1.1–14.4) (Table 2).

**Table 2:** Factors associated with patients being hospitalized for ILI and SRI associated with *Mycoplasma pneumoniae* infection, Klerksdorp and Pietermaritzburg, South Africa, June 2012–May 2015*

Factor	ILI, no. (%)	SRI, no. (%)	Univariate analysis			Multivariable analysis†	
OR (95% CI)	p value	aOR (95%CI)	p value
Age <5 y	11/27 (40.7)	38/73 (52.1)	1.6 (0.6–3.9)	0.317		7.1 (1.7–28.7)	**0.006**
Female sex	16/17 (59.3)	38/72 (52.8)	0.8 (0.3–1.9)	0.565			
Crowding, no. persons/room							
<2	18/27 (66.7)	49/72 (68.1)	Reference	NA			
3–4	7/27 (25.4)	19/72 (26.4)	1.0 (0.4–2.7)	0.996			
>5	2/27 (7.4)	4/72 (5.6)	0.7 (0.1–4.4)	0.734			
Duration of symptoms >4 d	7/26 (26.9)	37/69 (53.6)	3.1 (1.2–8.4)	**0.023**		3.8 (1.1–14.1)	**0.046**
HIV infection	2/23 (8.7)	32/62 (51.6)	11.2 (2.4–51.9)	**0.002**		23.8 (4.1–138.2)	**<0.001**
Any underlying medical condition‡	3/27 (11.1)	10/72 (13.9)	1.3 (0.3–5.1)	0.716			
Any viral co-infection§	13/27 (48.1)	47/73 (64.4)	1.9 (0.8–4.7)	0.144			

*Bold indicates statistical significance. aOR, adjusted OR; ILI, influenza-like illness; NA, not applicable; OR, odds ratio; SRI, severe respiratory illness. †Only variables found to be statistically significant (p<0.2) in univariate analysis were assessed in the multivariable model. ‡Underlying conditions defined as previously diagnosed chronic conditions including asthma, chronic lung diseases, cirrhosis or liver failure, chronic renal failure, heart failure, valvular heart disease, coronary heart disease, diabetes, burns, kwashiorkor or marasmus, nephrotic syndrome, spinal cord injury, seizure disorder, emphysema, and cancer, or history of immunosuppressive therapy or splenectomy. §Viruses tested were influenza types A and B, adenovirus, enterovirus, rhinovirus, human metapneumovirus, respiratory syncytial virus, and parainfluenza virus types 1–3.

### Rates of *M. pneumoniae* SRI Hospitalization

The mean annual rate of hospitalization for *M. pneumoniae* patients during 2013–2014 was 27.9 cases/100,000 population (95% CI 18.9–37.4) (Table 3). HIV-infected persons had 19.5 (95% CI 14.4–26.4) times greater odds of *M. pneumoniae*–associated SRI hospitalization (102.2/100,000 [95% CI 64.9–136.4) than did HIV-uninfected persons (14.9/100,000 [95% CI 10.3–19.0]). The highest rate was in patients <5 years old (220.0/100,000 [95% CI 121.0–314.8]).

**Table 3:** Incidence of hospitalization for *Mycoplasma pneumoniae*, by age group and HIV status, Klerksdorp and Pietermaritzburg, South Africa, January 2013–December 2014*

Age group, y	No. cases/100,000 population (95% CI)			Risk ratio (95% CI), HIV infected vs. uninfected
All patients	HIV-infected patients	HIV-uninfected patients
<1	220.0 (121.0–314.8)	594.8 (316.4–873.2)	216.7 (128.3–303.8)	2.8 (0.4–18.6)
1–4	53.9 (30.1–75.6)	961.7 (518.4–1391.6)	35.9 (21.1–50.2)	26.8 (14.4–49.8)
5–24	14.1 (9.4–18.6)	130.0 (81.1–173.8)	5.5 (3.9–7.1)	23.8 (13.4–42.4)
25–44	21.4 (15.0–27.6)	70.1 (46.3–95.1)	0 (0–46.7)	NA
45–64	26.2 (17.6–34.9)	118.7 (81.2–159.3)	4.7 (3.4–6.2)	25.6 (9.9–65.9)
>65	56.2 (37.5–74.2)	0 (0–234.1)	57.8 (41.3–74.9)	NA
<5	87.1 (60.2–114.0)	927.3 (621.3–1231.5)	72.3 (50.5–93.8)	16.1 (9.4–27.6)†
>5	20.5 (14.0–26.9)	91.3 (59.0–122.4)	6.3 (4.5–8.4)	21.6 (14.8–31.7)†
All	27.9 (18.9–37.4)	102.2 (64.9–136.4)	14.9 (10.3–19.0)	19.5 (14.4–26.4)†

*NA, not applicable. †Adjusted for age.

### Culture and Molecular Characterization

We obtained cultures for 11/77 (14%) *M. pneumoniae*–positive specimens. HRM macrolide susceptibility profiles were available for 43% (33/77) of *M. pneumoniae*–positive specimens, and all were macrolide-susceptible.

We obtained HRM analysis results for P1 genotyping for 36% (28/77) of *M. pneumoniae* PCR-positive specimens (*12*). *M. pneumoniae* were P1 type 1 (29% [8/28]), P1 type 2 (61% [17/28]), and a variant of P1 type 2 (11% [3/28]).

MLVA types were available for 51% (39/77) of PCR-positive samples. On the basis of a combination of tandem repeats at the 4 loci, 3 distinct types were present: 3/5/6/2 (17/39 [44%]), 3/6/6/2 (15/39 [38%]), and 4/5/7/2 (7/39 [18%]). The remaining 49% (38/77) could not be assessed because of the inability to determine the fragment size of >1 of the 4 variable-number tandem-repeat loci.

## Discussion

Overall, *M. pneumoniae* was detected in 1% of all patients (1.6% of SRI patients and 0.7% of ILI patients). Among *M. pneumoniae* patients, young age (<5 years) and HIV infection were associated with severe disease. *M. pneumoniae* strains were susceptible to macrolides and represented 3 P1 types. The higher detection rate of *M. pneumoniae* in periods 1 and 2 of the study suggests that there might be periodicity in *M. pneumoniae* infection in South Africa.

The prevalence of *M. pneumoniae* varies depending on whether a study was performed during an endemic or epidemic year, the laboratory detection method used, or the study participants (*3*). During 2010–2012, an epidemic of *M. pneumoniae* occurred in Denmark, England, Wales, Sweden, Finland, and Germany, with detection rates ranging from 12% to 17% (*23*–*26*). In France, detection rates of *M. pneumoniae* ranged from 2% to 10% during a 5-year period among outpatients with an acute respiratory illness (*5*). However, higher detection rates of 27%–30% among children with community-acquired pneumonia have been reported in the United States and Finland and up to 60% among hospitalized adults with pneumonia in Japan (*3*,*4*,*27*). Jain et al. reported that, among hospitalized children in the United States, *M. pneumoniae* was the most common bacterial cause of community-acquired pneumonia, accounting for 8% of cases (*28*), and among hospitalized adults in the United States, *M. pneumoniae* was identified in ≈2% of cases (*29*). The prevalence differences in our study compared with other studies might be attributable to a difference in enrollment criteria, the age group of participants, and HIV prevalence among the participants.

Despite having low detection rates, *M. pneumoniae* was significantly associated with illness. The fraction of illness attributable to *M. pneumoniae* in patients testing positive was 80.7% in ILI patients, 90.1% in SRI patients with *M. pneumoniae* detected on nasopharyngeal specimens only, and 93.9% in SRI patients with *M. pneumoniae* detected on both nasopharyngeal and sputum specimens. These results suggest that *M. pneumoniae* can be considered a likely pathogen when detected in patients with ILI or SRI, regardless of specimen type.

We did not observe a distinct seasonal pattern of *M. pneumoniae*. Several more years of surveillance of *M. pneumoniae* is essential to elucidate seasonality in our setting. However, a significant difference was noted in the detection rate over the 3 study periods. Layani-Milon et al. reported that, during a 5-year period (1993–1997), rates of *M. pneumoniae* disease varied monthly and yearly and *M. pneumoniae* occurred in epidemic cycles (*5*). Furthermore, in a serologic study in Johannesburg, South Africa, during 1969–1975, the periodicity of *M. pneumoniae* was shown to peak in 3-year intervals (*7*). During period 1 of our study, a cyclical epidemic of *M. pneumoniae* was probably occurring, and this epidemic reached its nadir in period 3 of the study. Longer study periods are required to evaluate the cyclical nature of *M. pneumoniae*.

In our study, a large proportion (65%) of *M. pneumoniae* patients were co-infected with a respiratory virus, of which rhinovirus and adenovirus were the most common. Similarly, Lieberman et al. reported that, in 65% of *M. pneumoniae* patients, >1 additional pathogens were detected, of which *Streptococcus pneumoniae* (43%) and *Legionella* spp. (15%) were most frequently detected (*30*).

We found that young age (<5 years) and HIV infection among *M. pneumoniae*–positive persons were independently associated with severe disease. HIV association with *M. pneumoniae* disease was reported in a study conducted in India during 2004–2007 (*31*). The incidence rate of *M. pneumoniae* in South Africa was 28 cases/100,000 population, with the highest incidence occurring in children <5 years old at a rate of 87 cases/100,000 population. We observed a greater disease prevalence among HIV-infected patients than HIV-uninfected patients. Other studies have reported incidence rates ranging from 180 to 1,290 cases/100,000 population/year (*3*,*5*,*32*). Studies have shown variability in detection rates among different age groups, especially in *M. pneumoniae*–endemic areas, where *M. pneumoniae* has occurred predominantly among children <5 years old (*33*,*34*).

A lack of consensus exists regarding the preferred specimen type for the identification of *M. pneumoniae* (*35*,*36*). We observed a significantly higher detection rate of *M. pneumoniae* in sputum compared to nasopharyngeal specimens, similar to results reported by Dorigo-Zetsma et al. (*37*) and Räty et al. (*38*). Although we detected a higher rate in sputum, nasopharyngeal specimens remain the preferred specimen type for surveillance because collecting a nasopharyngeal specimen is less invasive. In addition, a positive result on a nasopharyngeal specimen is a good indicator of disease as indicated by the AF.

During the *M. pneumoniae* epidemic that occurred in Europe during 2010–2012, P1 type 1 was dominant (*24*,*39*). In our population, P1 types 1 and 2 were circulating at equal frequencies. Likewise, in the United States, during an 8-year period (2006–2013) both P1 types were co-circulating (*40*). In China, during 2009–2011, P1 type 1, type 2, and variants of type 2 were identified; however, a higher frequency of type 1 compared with the other P1 types was observed (*41*). Continued surveillance is important to identify longer-term trends in *M. pneumoniae* strain prevalence in South Africa.

By using the 4-loci MLVA typing scheme and nomenclature, we identified 3 distinct MLVA types (3/6/6/2, 3/5/6/2, and 4/5/7/2), which are the same types circulating in the United States, Kenya, Guatemala, Egypt, Denmark, and Canada (*19*,*20*,*40*,*42*). However, the predominance of MLVA type 3/5/6/2 in our study was different to what has been previously described elsewhere in the world. The predominant MLVA type circulating in numerous countries during 1962–2013 was MLVA type 4/5/7/2 (*13*,*20*,*40*). Although no correlations of strain type with disease severity or outcomes have been established, these typing methods are useful for monitoring trends over time and during outbreak investigations.

Macrolide resistance of 17% was documented in *M. pneumoniae* in Japan during 2000–2003 (*43*), with even higher rates of up to 90% reported in China (*44*). In Germany, 1.2% and 3% of *M. pneumoniae* found in respiratory tract specimens were resistant to macrolides during 2003–2008 and 1991–2009, respectively (*45*). In a US study, macrolide resistance was reported for ≈3% of *M. pneumoniae* cases in patients hospitalized with community-acquired pneumonia (*46*). However, other studies have reported macrolide resistance of 10%–13% in sporadic and outbreak specimens in the United States (*40*,*47*). Resistance in Europe and the United States remains low relative to Asia, possibly because of the restricted availability of antimicrobial drugs. We did not identify macrolide resistance among the isolates in our study, and therefore macrolide treatment is probably effective against *M. pneumoniae* in our setting. However, excessive use of macrolides should be discouraged, given that in Japan inappropriate use of macrolides was shown to increase the likelihood of the organism developing mutations in the 23S rRNA gene (*11*). Therefore, identification of the etiologic cause of infection and its appropriate treatment are essential. In South Africa, first-line treatment for community-acquired pneumonia is penicillin (*48*). In severely ill persons or those in whom atypical pneumonia is suspected, macrolides are administered. A limitation of our study is that treatment data for patients with *M. pneumoniae* were limited.

We performed molecular characterization for samples collected during periods 1 and 2 of our study. Most of the positive specimens were obtained during these 2 periods, and results from these periods can be inferred for period 3 because no intervention was implemented. We obtained a low yield of isolates and were unable to determine the macrolide susceptibility trait and strain type for a proportion of specimens, most likely because of a low bacterial load in the specimen, which might have affected the ability to detect resistance particularly if a low prevalence of macrolide-resistant *M. pneumoniae* strains exists in South Africa.

We have shown that, although the *M. pneumoniae* detection rate was low, *M. pneumoniae* detection is probably associated with illness, underscoring the need for testing, especially among patients at higher risk for severe disease. Such testing would result in an earlier diagnosis and improved management. Our study provides baseline data that can be used for future surveillance programs to better understand *M. pneumoniae* epidemiology in South Africa.
